# Assessing Vaccine Coverage and Timeliness in Bamako, Mali after the Introduction of Rotavirus Vaccine: A Modified Immunization Cluster Survey

**DOI:** 10.4269/ajtmh.21-0148

**Published:** 2021-10-04

**Authors:** Anna Roose, Uma Onwuchekwa, Milagritos Tapia, Samba Sow, T. Christopher Mast, Karen Kotloff

**Affiliations:** ^1^ Center for Vaccine Development and Global Health, University of Maryland School of Medicine, Baltimore, Maryland;; ^2^ Centre for Vaccine Development, Bamako, Mali;; ^3^ Department of Pediatrics and Department of Medicine, University of Maryland School of Medicine, Baltimore, Maryland;; ^4^ Department of Pharmacoepidemiology, Merck & Co., Inc., North Wales, Pennsylvania

## Abstract

Vaccine coverage and timeliness are critical metrics for evaluating the performance of immunization programs. Following the introduction of rotavirus vaccine in Bamako, Mali, we conducted two cluster surveys spaced approximately 1 year apart to evaluate these metrics among children 9 to 20 months of age. Using the child’s immunization card or the medical record at the center of administration, each selected child’s immunization status was determined at 9 and 12 months of age. Deviations from the WHO-recommended immunization schedule were described by the median delay and fraction of children receiving doses outside of recommended age ranges. Overall, 1,002 children were enrolled in the two surveys combined; 80.1% of children born 7 to 12 months after introduction (survey 1) received three doses of pentavalent rotavirus vaccine (ROTA3) by 9 months of age, which increased to 86.1% among children born 17 to 26 months after introduction (survey 2). Concomitantly, coverage with the third dose of diphtheria-pertussis-tetanus-containing vaccine (DPT3) by age 9 months was 86.5% (survey 1) and 88.9% (survey 2); by age 12 months, 61.3% and 72.4% of children, respectively, had received all scheduled immunizations. The median delay in ROTA3 and DPT3 administration were similar at about 3.4 weeks. Within 3 years of introduction, coverage of rotavirus vaccine among Bamako infants achieved coverage similar to DPT3 and is approaching the Global Vaccine Action Plan goal of 90% coverage by 2020. However, timeliness of coverage remains a concern.

## INTRODUCTION

Since the introduction of the WHO’s Expanded Program for Immunization (EPI) in 1974 and the creation of Gavi, the Vaccine Alliance in 2000, the availability of immunizations to children in low- and middle-income countries has greatly improved.^1^^–^^3^ Nevertheless, significant global inequalities in vaccine coverage and access persist. The African region, where 45–50% vaccine-preventable morbidity and mortality occurs,^4^ has the lowest coverage of core childhood immunizations of all WHO regions. In this region, estimated coverage for the third dose of diphtheria-pertussis-tetanus containing vaccine (DPT3) was only 72% in 2016.^5^ During the decade since WHO recommended introduction of rotavirus vaccine into all national immunization programs, only three-quarters of sub-Saharan African countries have followed suit, even though most diarrheal deaths occur in sub-Saharan Africa and most are attributable to rotavirus.^6^^–^^8^ Moreover, implementation has lagged in countries bearing the highest rates of rotavirus-associated mortality.^9^

Even in settings with high vaccine coverage, deviations in the timing of vaccination may interfere with vaccine effectiveness. If administered too early, factors such as interference from maternal transplacental antibody and immunological immaturity may prevent an effective immune response,^10^^–^^12^ whereas delayed administration may leave a child vulnerable to infection during the ages where attack rates are high.^13^^,^^14^ A 2009 review of the timing of vaccine administration in 45 countries found that the median delay was 6.2 weeks for DPT3, and among West African nations, median coverage of DPT3 by 6 months of age was only 30%.^15^ Assessment of immunization programs at the country and sub-country level, as well as identification of points at which performance is inadequate, is essential to improving the effectiveness of immunization coverage.

Mali is a low-income sub-Saharan African nation with the second highest under five child mortality rate globally.^16^ The vaccine schedule recommended for infants by the Malian EPI is as follows: Bacillus Calmette-Guerin (BCG) and oral polio vaccine (OPV) at birth; OPV, pentavalent vaccine (containing antigens for DTP, hepatitis B, and *Haemophilus influenzae* type b, referred to here as “DPT”), pentavalent rotavirus vaccine (ROTA),and pneumococcal conjugate vaccine (PCV) at 6, 10, and 14 weeks; and measles and yellow fever vaccines at 9 months of age. In 2017, an estimated 92% of Malian infants received BCG and 86% received the first dose of DPT (DTP1), whereas rates for three doses of DTP3, hepatitis B, pneumococcal conjugate vaccine (PCV3), and measles vaccine were only 60–68%.^17^ Coverage rates in Bamako, the capital city, generally exceed national estimates.^18^^,^^19^ Mali added RotaTeq^®^ (Rotavirus Vaccine, Live, Oral, Pentavalent; Merck Vaccines, Whitehouse Station, NJ) to its immunization schedule in early 2014; one year later, the 2015 Multiple Indicators Cluster Survey found that 23.4% of infants nationally and 73.1% of infants in Bamako had received all three doses of the series (ROTA3).^18^ Changes in coverage over the ensuing years was not known at the time of this study.

A modified formulation of ROTA (RV5mp) was developed with stability at 37°C for 7 days and an expiry extended to 36 months when stored at 2–8°C.^20^ As part of plans to license and introduce RV5mp, among infants living in Bamako, efforts were initiated to evaluate the safety and effectiveness of RV5mp. This study required 2 years of baseline data on vaccine coverage with the marketed formulation of the vaccine (RV5) that was in use at the time. We conducted two vaccine coverage surveys approximately 1 year apart, in 2015 and 2016, focusing on the uptake of rotavirus vaccine and the timeliness of the administration of vaccines in the first year of life.

## METHODS

### Study population and data collection.

The primary aim of the study was to determine the rate of rotavirus vaccine coverage during the preceding 12 months using serial two-stage cluster surveys of a random sample of infants and children residing in Bamako, Mali. Because rotavirus vaccine should not be administered after 6 months of age, we chose to assess coverage rates at the time the children were age 9 months among a sample of children 9 to 20 months old to minimize the well-documented risk of card loss.^18^^,^^19^ As secondary aims, we assessed the 9-month coverage of other routine vaccines scheduled to be completed at 14 weeks, and the coverage of vaccines scheduled to be administered at 9 months of age among those 12 months or older. Birth dose of OPV was not recorded, and inactivated polio vaccine and meningococcus A vaccine had not yet been introduced. Finally, we evaluated the timeliness of vaccination, describing the median age and median delay in dose administration from its scheduled time, as well as the fraction of children immunized outside of recommended windows.

Eligible children resided in Bamako for at least 6 months before the survey and were between 9 and 20 months of age on the day that the survey started. Two surveys were conducted in 2014–2016: survey 1 took place from March 21, 2016 to April 25, 2016, which assessed coverage among children born between July 21, 2014 and June 16, 2015, and survey 2 was conducted from February 20, 2017 to March 27, 2017, assessing coverage among children born between June 21, 2015 and May 31, 2016. Written documentation of vaccination was required, sought first from the child’s immunization or health card. If the card could not be located after three visits to the household, records were sought from registers at the health center where the child was vaccinated. Birthdates recorded on the child’s immunization card were validated using the maternal or child health card whenever possible. A mother’s report that the child had never received immunizations was accepted as evidence of no vaccination. Dates of immunization were recorded on case report forms and a photograph of the immunization card or healthcare registry was obtained for quality control purposes.

The surveys were approved by the Institutional Review Board of the University of Maryland, Baltimore and by the Ethics Committee of the Faculté de Médecine, de Pharmacie et d’Odonto-Stomatologie, University of Mali. A parent or guardian gave consent for the participation of each child before any research activities were conducted.

### Survey design.

Surveys methods were adapted from the WHO’s 2005 immunization coverage cluster survey reference manual,^21^ modified because household listings were not available. Data from the most recent census of Bamako, which was completed in 2009 by the Mali National Institute of Statistics (INSTAT), were used to establish the sampling frame for the survey.

Bamako is administratively divided into six communes, each of which was subdivided into 1,047 “enumeration sections” for the purpose of the 2009 census. Theses enumeration sections were modified in two ways to create the primary sampling units (PSUs) for our survey. First, the size of the population residing in each enumeration section at the time of the survey was estimated using commune-specific growth rates provided by INSTAT, which ranged from 2.2% to 7.5% annually. The annual number of children in a section was estimated by $u_{y}=u_{2009}\times {\left(1+g\right)}^{y-2009}$, where $u_{2009}$ is the number of children in the section in 2009, *y* is the year of the survey, and *g* is the annual growth rate in the commune where the section is located. Second, to create clusters of approximately 50 age-eligible children, adjacent enumeration sections were further combined or divided as needed; these clusters constituted the PSU for the survey.

**Figure 1.**
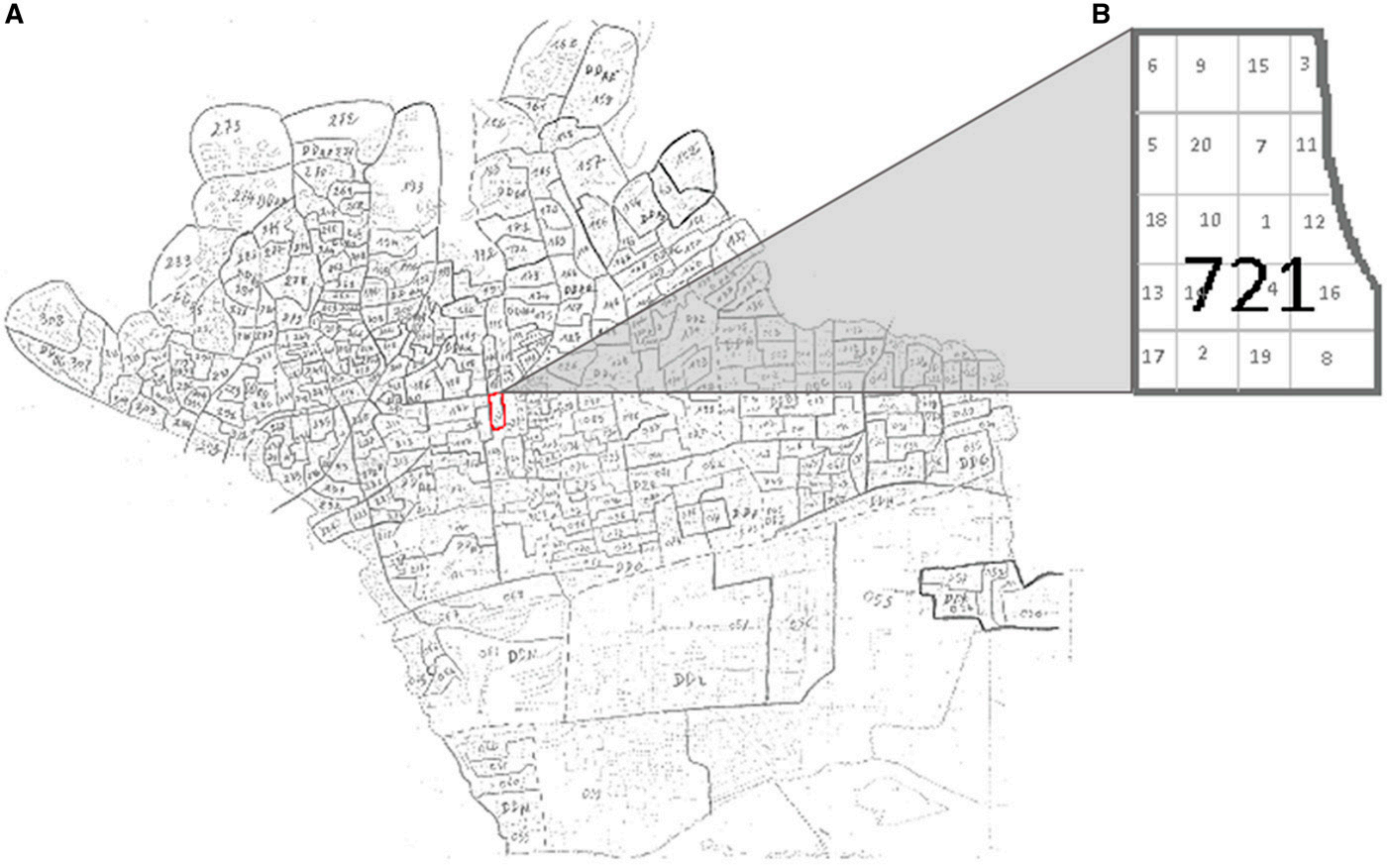
Example map used for identifying survey clusters. Clusters were identified and drawn on maps of the Bamako communes (**A**, commune 1 shown here). Each cluster was divided into a grid of 20 cells (**B**).

Clusters were mapped for survey interviewers (Figure 1A). For each survey, a new list of 60 clusters was randomly selected using the “sample.int” function from the R package *base* version 3.5.1. for possible inclusion, from which the first 50 were included in the survey and the remaining 10 served as back-ups. Clusters were sampled without replacement and with probability proportional to size. Each cluster was then gridded into 20 subunits, which served as the secondary sampling units. Each subunit was assigned a unique number ranging between 1 and 20 (Figure 1B). The numbers 1 to 20 were ordered randomly using Microsoft Excel, and fieldworkers visited the subunits sequentially to identify one participant in each subunit until a total of 10 participants were enrolled in each cluster.

A household was defined as individuals who share a cooking fire.^22^ Households within a gridded subunit were selected by locating the house in the southeast-most corner of the subunit and progressing door-to-door down one side of the street and turning right at the end of each block of the subunit until an eligible child was identified whose parent provided informed consent. If no child could be identified within the subunit, the interviewer looked for a child in the next subunit. If there were multiple households within a single dwelling, a household was selected at random by enumerating the households on slips of paper and drawing one number, and if multiple children were eligible within a household, one of the children was selected by numbering the children and randomly ordering those numbers in Microsoft Excel. Approximately one in 188 and one in 198 age-eligible children residing in Bamako were selected for the 2016 and 2017 surveys, respectively.

## STATISTICAL METHODS

Vaccine coverage was calculated separately for each survey to detect differences in rotavirus vaccine uptake between surveys and together to provide period-specific coverage estimates. Because clusters were selected with probability proportional to size, it was not necessary to weight the individual responses to represent the larger 9 to 20 month population of Bamako. Vaccine coverage was described using proportions with a binomial 95% confidence interval. The completion of scheduled immunizations was analyzed at 9 and 12 months of age, i.e., the child’s birth date and immunization dates for each vaccine were used to determine whether the child had been immunized by 9 and 12 months of age. Children were deemed “fully vaccinated” at the 9- and 12-month timepoints if they had received all vaccinations recommended by the Malian EPI prior to each timepoint.

Pooling the two surveys, we used the Kaplan–Meier method to assess timeliness of dose administration. Children who had not received the vaccine dose by 12 months of age were censored. The median age at administration and the median delay from the scheduled administration age was calculated for each dose, as was the fraction of children vaccinated before and after the WHO-recommended window.^15^ Additionally, we calculated the fraction of children who did not receive all recommended 6-week, 10-week, or 14-week doses on the same day.

Study data were entered into a Microsoft Access database at the time of interview, followed by quality review, cleaning, and analysis using the statistical software platform R version 3.3.2.^23^

## RESULTS

**Figure 2.**
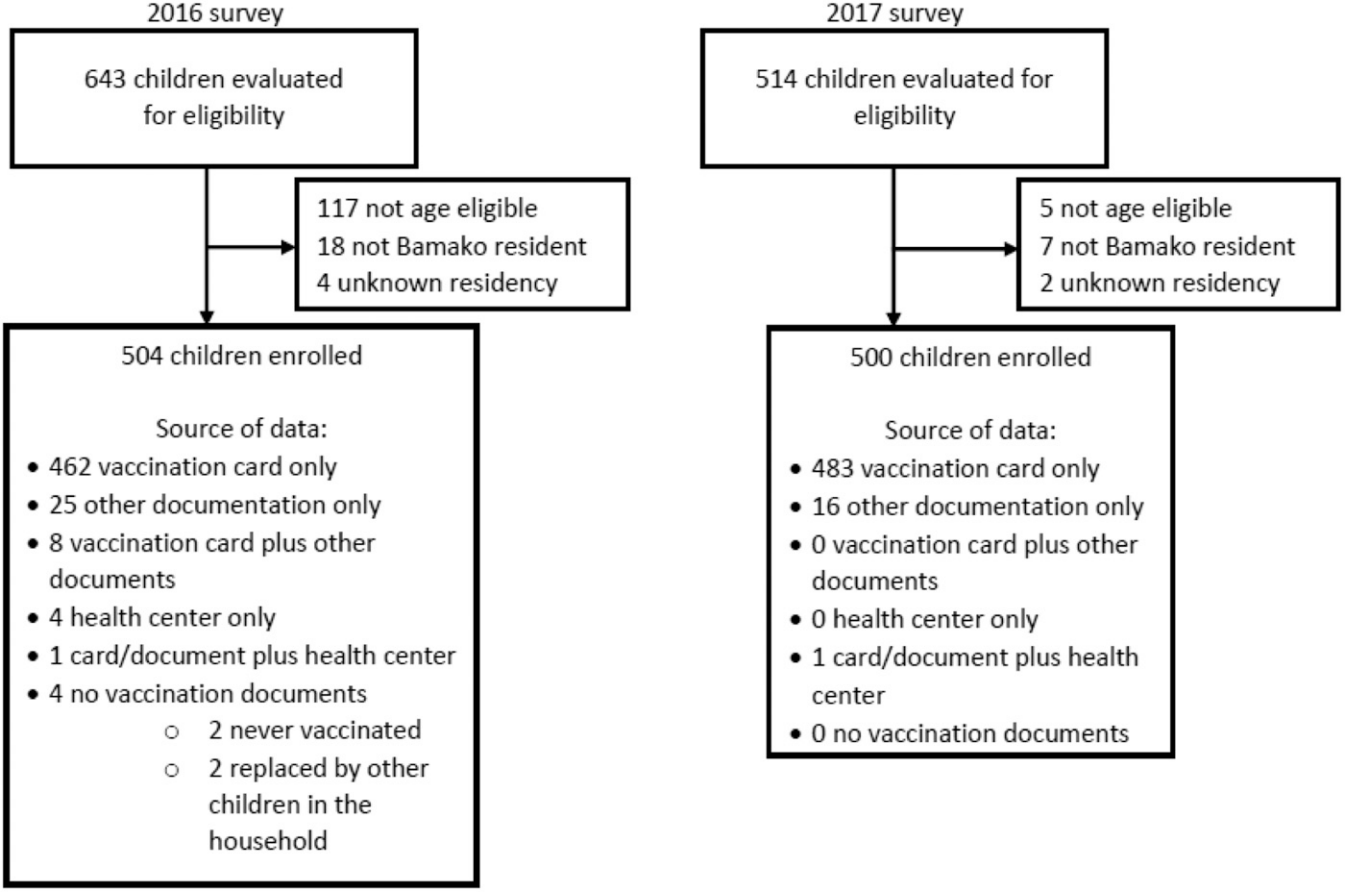
Consort diagram of enrollment in 2016 and 2017 surveys and contribution to overall analysis.

In the two vaccine coverage surveys combined, 1,004 age-eligible children were invited to enroll, 504 in the 2016 survey and 500 in the 2017 survey (Figure 2). For one cluster in one survey, only nine eligible children were identified after visiting each of the 20 subunits. A 10th child was recruited from a 51st cluster. Additionally, two children whose vaccination cards were not available were replaced with age-eligible children within the same household. No caregiver refused to participate in the survey. Demographic characteristics for surveyed children are shown in Table 1. Across the two surveys, 804 children (80.2%) were 12 months of age or older. The mean age of participants was 15.0 months (SD 3.1). The vaccination card was available for 95% of children surveyed. Of the 47 children who did not have a vaccination card available, 41 had information retrieved from another written record (i.e., a health card), four had records available at the immunizing clinic, two provided information from a record other than the vaccination card and from the health center, and two had never been vaccinated. For eight children, vaccination cards were supplemented by other records.

**Table 1:** Demographic characteristics of surveyed infants

	2016 Survey (*N* = 502)	2017 Survey (*N* = 500)
Male children, n (%)	256 (51%)	281 (56%)
Mean age in months, range	15.0 (10–20)	14.8 (9–20)
Maternal education, n (%)		
No formal schooling	217 (43%)	225 (45%)
Primary education	176 (35%)	159 (32%)
Secondary education	92 (18%)	72 (14%)
Postsecondary education	18 (4%)	36 (7%)
Vaccination card available, n (%)	471 (93%)	483 (100%)

Point estimates of vaccine coverage in 2017 were generally higher than in 2016, although binomial confidence intervals overlap (Table 2). In both surveys, receipt of BCG was nearly universal (> 99% of children). The third dose of diphtheria-pertussis-tetanus-containing vaccine coverage was 86.5% in 2016 and 88.9% in 2017. Among the primary series administered at 6, 10, and 14 weeks, coverage was lowest for rotavirus vaccine in survey 1 (80.1% at 9 months of age) and survey 2 (84.3% at 9 months of age). Coverage with measles and yellow fever vaccines by 12 months of age was substantially lower than coverage with vaccines administered at younger ages (Table 2). By 12 months of age, 79.7% (642/806) of children had completed BCG, DPTCV3, OPV3, and ROTA3 series, but only 70.5% (568/806) had received yellow fever and measles vaccine (*P* < 0.001, two-proportion Z-test). In both surveys, it was rare (< 1%) for a child to be completely unvaccinated, but full vaccine coverage by 12 months of age was only 61.3% in 2016 and 72.4% in 2017.

**Table 2:** Proportion of children (95% CI) receiving EPI immunization per vaccination card, by survey year

	2016 Survey	2017 Survey
Dose	*N* = 502	*N* = 500
Coverage at 9 months:		
BCG	99.2 (98.0–99.8)	99.4 (98.3–99.9)
DPT1	96.8 (94.9–98.2)	97.0 (95.1–98.3)
DPT2	92.6 (89.9–94.7)	92.8 (90.2–94.9)
DPT3	86.5 (83.2–89.4)	87.3 (84.1–90.1)
OPV1	93.6 (91.1–95.6)	97.2 (95.3–98.5)
OPV2	90.4 (87.4–92.8)	92.4 (89.7–94.6)
OPV3	84.9 (81.5–88.0)	86.7 (83.4–89.6)
PCV1	92.0 (89.3–94.2)	96.2 (94.1–97.7)
PCV2	87.6 (84.4–90.4)	91.8 (89.0–94.0)
PCV3	80.5 (76.8–83.9)	86.3 (83.0–89.2)
ROTA1	93.0 (90.4–95.1)	93.9 (91.4–95.9)
ROTA2	87.4 (84.2–90.2)	89.0 (86.0–91.7)
ROTA3	80.1 (76.3–83.5)	84.3 (80.8–87.4)
Coverage at 12 months:		
Measles	67.4 (62.6–72)	75.0 (70.5–79.1)
Yellow fever	67.9 (63.1–72.5)	75.1 (70.5–79.2)
Completely unvaccinated	0.5 (0.1–1.8)	0.2 (0–1.4)
Fully vaccinated*	61.3 (56.3–66.1)	72.4 (67.8–76.7)

BCG = Bacillus Calmette-Guerin vaccine; CI = confidence interval; DPT = diphtheria, pertussis, and tetanus-containing vaccine, administered in combination with *Haemophilus influenzae* type b conjugate vaccine and hepatitis b vaccine as pentavalent vaccine; OPV = oral polio vaccine; PCV = pneumococcal conjugate vaccine; rota = rotavirus vaccine.

* Fully vaccinated children received all recommended immunizations.

**Figure 3.**
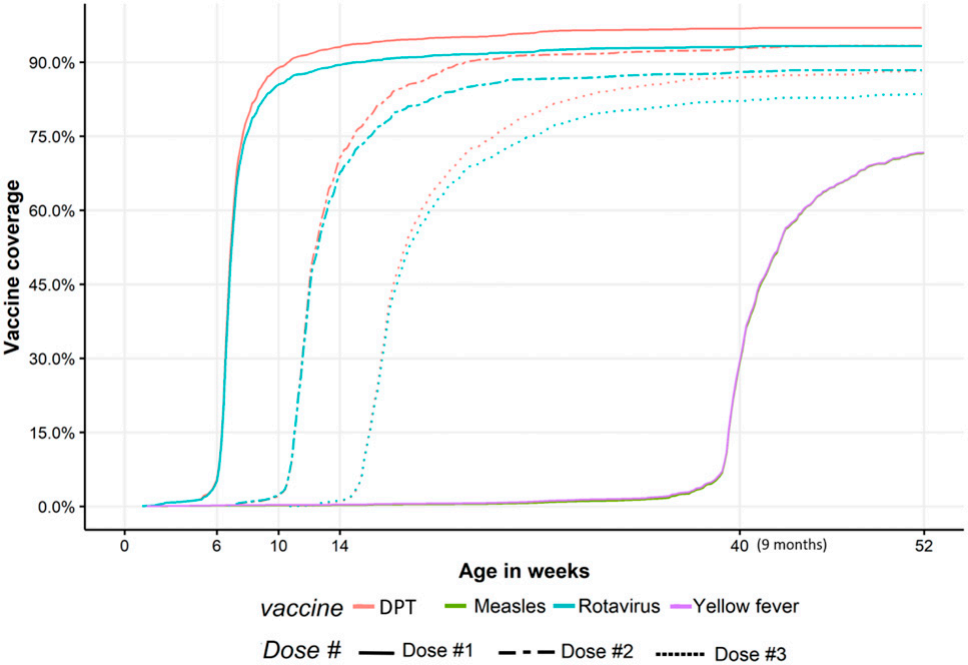
Age of diphtheria, pertussis, tetanus-containing vaccine (DPT), rotavirus, measles, and yellow fever vaccine administration in Bamako, Mali. Doses of DPT and rotavirus vaccine are scheduled to be administered at 6, 10, and 14 weeks. Measles and yellow fever are scheduled to be administered at 9 months of age and overlapped perfectly in our data. This figure appears in color at www.ajtmh.org.

Figure 3 shows the plot of the Kaplan–Meier estimator of vaccine coverage for each dose of the DPT and rotavirus vaccine series. In general, DPT and ROTA doses were administered according to the same schedule, with rotavirus vaccine administration very slightly delayed relative to DPT vaccine. For all immunizations, administration became progressively more delayed as children proceeded through the primary 6-, 10-, and 14-week immunization series, with half of children receiving their 14-week doses at least 3.4 weeks late (Table 3). The median delays for measles and yellow fever doses were 1.6 and 1.4 weeks, respectively. Early administration of doses was uncommon for all vaccines. Late administration was most common among the 14-week doses, with 9.9–13.2% of children receiving these doses after 6 months of age. 86.9%, 84.8%, and 82.5% of children received all 6-week, 10-week, and 14-weeks doses together. Nearly all children (98.2%) who received recommended 9-month vaccines received both vaccines on the same day.

**Table 3:** Median age (range*) and timeliness of dose administration

	Birth dose	6-week doses				10-week doses				14-week doses				9-month doses (39 weeks)	
	BCG	OPV1	DPT1	PCV1	ROTA1	OPV2	DPT2	PCV2	ROTA2	OPV3	DPT3	PCV3	ROTA3	Measles	Yellow Fever
Median age at dose in weeks (range)	1.6	6.9 (1.1–41.4)	6.9 (1.1–41.4)	6.9 (1.1–41.4)	6.9 (1.1–41.4)	12.1 (6.7–52.7)	12.1 (6.7–52.7)	12.1 (6.7–52.7)	12.1 (6.7–52.7)	17.4 (10.7–59.6)	17.4 (10.7 −52.3)	17.7 (10.7–69.4)	17.4 (10.7–70.4)	40.6 (1.4–76.7)	40.4 (1.4–76.7)
Median delay in weeks (range)	1.6	0.9 (−4.9–35.4)	0.9 (−4.9–35.4)	0.9 (−4.9–35.4)	0.9 (−4.9–35.4)	2.1 (−3.3–42.7)	2.1 (−3.3–42.7)	2.1 (−3.3–42.7)	2.1 (−3.3–42.7)	3.4 (−3.3–45.6)	3.4 (−3.3–38.3)	3.7 (−3.3–55.4)	3.4 (−3.3–56.4)	1.6 (−37.6–37.7)	1.4 (−37.6–37.7)
% Inter-dose interval < 4 weeks	–	–	–	–	–	1.9	2.0	2.4	1.9	1.2	0.9	0.9	1.0	–	–
Recommended administration ages^†^	Birth– 8 weeks, *N* = 1,004	4 weeks–2 months, *N* = 1,004				8 weeks–4 months, *N* = 1,004				12 weeks–6 months, *N* = 1,004				9 months – 12 months, *N* = 806	
% Vaccinated before minimum age	0	1.5	1	1.2	1.1	0.9	0.9	0.9	0.9	0.1	0.1	0.1	0.2	6.1	6.5
% Vaccinated after maximum age	2.6	12.5	13.1	14.6	13.1	9.8	10.1	12.6	10.1	9.9	10.3	13.2	10.7	3.4	3.3
% Vaccinated after 6 months of age	0.4	1.0	1.2	1.2	1.4	2.1	2.4	2.8	2.3	9.9	10.3	13.2	10.7	–	–
% Received all doses together (on time)	NA	86.9				84.8				82.5				98.2	

BCG = Bacillus Calmette-Guerin vaccine; DPT = diphtheria, pertussis, and tetanus-containing vaccine, administered in combination with *Haemophilus influenzae* type b conjugate vaccine and hepatitis b vaccine as pentavalent vaccine; OPV = oral polio vaccine; PCV = pneumococcal conjugate vaccine; ROTA = pentavalent rotavirus vaccine.

* Negative values occur because some children received vaccine doses before the recommended age.

† Ranges were adapted from published criteria.^15^

Pooled coverage survey results for comparison with recent Multiple Indicator Cluster Surveys (MICS) and demographic and health survey (DHS) results are shown in Table 4. Results were generally similar to previously reported coverage results, although rates of complete coverage were higher in the present surveys (66.7% versus 40.9–47.8%) (Table 4). Notably, we obtained much higher rates of vaccination card review (96.5% versus 45.9–56.0%) (Table 4).

**Table 4:** Comparison of methods and results for recent vaccine coverage surveys in Bamako, Mali

Survey	2016 and 2017 Bamako-wide surveys	2012–2013 Demographic and health survey^18^	2015 Multiple indicators cluster survey^19^	2018 Demographic and health survey^24^
Age surveyed	9–20 months	12–23 months	12–23 months	12–23 months
Selection of survey participants	PSUs were contiguous enumeration sections that were combined to contain at least 50 children 6–23 months; secondary sampling units were 20-cell grid over the PSU. The first child identified in the cell was interviewed	PSUs were enumeration sections; secondary sampling units were selected at random from a household listing	PSUs were enumeration sections; secondary sampling units were selected at random from a household listing	PSUs were enumeration sections; secondary sampling units were selected at random from a household listing
Assessment of vaccine status	Reviewed vaccination card, returning to household if the card was unavailable. If the card had been lost, destroyed, or continued to be unavailable, surveyors traveled to the EPI center to review records. Maternal report was accepted only for the non-vaccination of the child	Reviewed the vaccination card, and accepted the mother’s statements when the card was unavailable or did not exist	Reviewed the vaccination card, and accepted the mother’s statements when the card was unavailable or did not exist	Reviewed the vaccination card, and accepted the mother’s statements when the card was unavailable or did not exist
Number of children ≥ 12 month surveyed	804	200	357	200
Vaccine coverage by 12 months of age				
BCG	99.3%	95.0%	93.6%	96.6%
OPV0	–†	84.9%	89.5%	92.8%
DPT1	97.6%	89.7%	89.9%	93.3%
OPV1	96.4%	91.8%	84.6%	87.5%
PCV1	95.0%	–‡	86.0%	92.4%
ROTA1	94.5%	–‡	83.9%	92.7%
DPT2	94.3%	83.7%	84.3%	84.4%
OPV2	92.6%	83.4%	81.3%	83.6%
PCV2	91.0%	–‡	80.4%	84.8%
ROTA2	89.6%	–‡	79.5%	85.1%
DPT3	89.1%	74.1%	80.3%	82.0%
OPV3	88.1%	57.8%	50.1%	54.2%
IPV				64.0%
PCV3	85.9%	–‡	74.3%	82.3%
ROTA3	84.4%	–‡	73.1%	81.8%
Measles	71.2%	79.6%	78.9%	83.5%
Yellow fever	71.5%	37.1%	75.9%	76.8%
% Complete coverage*	66.7%	45.7%	40.9%†	47.8%
% Complete noncoverage	0.4%	3.5%	6.0%	2.6%
Rate of card availability	96.5%	45.9%	55.2%	56.0%

BCG = Bacillus Calmette-Guerin vaccine; DPT = diphtheria, pertussis, and tetanus-containing vaccine; IPV = inactivated polio vaccine; OPV = oral polio vaccine; PCV = pneumococcal conjugate vaccine; PSU = primary sampling units; ROTA = pentavalent rotavirus vaccine.

* Defined as covered with BCG, one dose of measles vaccine, three doses of polio, PCV (once introduced), and DPT/pentavalent vaccine by 12 months of age.

† Information on birth dose of OPV was not collected.

‡ PCV and ROTA had not yet been introduced in Mali.

## DISCUSSION

Our two surveys of vaccine coverage among Bamako infants over a 22 consecutive month period found that although the coverage for the newly introduced ROTA was slightly lower than coverage of other coadministered vaccines, rates were high and improving both in absolute terms and relative to DPT year-over-year. Card availability and coverage estimates in our surveys were generally higher than those reported during the 2012–2013 DHS, the 2015 MICS, and the 2018 DHS (Table 4). The increased vaccination card availability in our study was a notable difference from previously published results. Maternal report was accepted as evidence of vaccination in the DHS and MICS surveys but not in our survey, which makes the accuracy of our higher vaccination rate data more likely. We attribute our field team’s success in retrieving immunization cards to the use of local interviewers who were trained to visit households when mothers of young children are likely to be available (e.g., during meal preparation), to community sensitization activities conducted immediately before the survey began, to the requirement that the interview team make multiple visits to households and clinic visits to retrieve records, and to the recent requirement that immunization cards be presented for children enrolling in public schools in Bamako. Our interviewers learned that head of the household often control access to vaccination cards, and arranged with the families to conduct the interview when the card would be accessible. Finally, we interviewed a younger age group than is typical for coverage surveys to maximize the likelihood that the card would be found.

We also explored the timeliness of the EPI immunizations among surveyed infants. We observed that immunization delays became more common as children aged, and that over 10% of children received their 14-week doses after 6 months of age. All vaccines scheduled to be delivered together appeared to be similarly delayed. Delayed immunization can compound the overall vulnerability of children to vaccine-preventable infections in resource-poor settings where reduced vaccine effectiveness is observed.^25^^–^^28^ Nonetheless, the 3.4-week delay beyond the age at which DPT3 was scheduled in Bamako was shorter than the 6.2-week median previously reported across 45 countries.^15^

We observed that 17.5% of children did not receive all four scheduled doses (i.e., DPT, PCV, OPV, and ROTA) recommended for the 14-week immunization concomitantly, suggesting that all routinely coadministered vaccines may not be available to a small but meaningful fraction of children when they present to EPI centers. These instances constitute missed opportunities for immunization and may contribute to late and incomplete coverage. It is notable that the EPI delivery system was able to quickly introduce a newly available rotavirus vaccine and to rapidly achieve coverage rates that were similar to vaccines that had been available for many years.

Our study has several limitations. Our surveys differed methodologically from the DHS and MICS surveys in ways that may contribute to differences in our findings and hamper comparability. First, although all three surveys used data from Mali’s 2009 national census to identify clusters, our survey updated the estimates of PSU size using commune-specific growth rates from INSTAT. We believe this resulted in the selection of PSUs that were more representative of the Bamako population, which has grown much more quickly in some communes than others. Because we were not able to use or create household listings within the PSUs, household selection in our second stage sampling may have been vulnerable to bias because selection was not totally random, although a systematic source of bias was not identified.

Our surveys indicate that the EPI in Bamako has achieved high coverage with three doses DTP, with most doses being timely. Mali can be recognized as an early introducer of rotavirus vaccine in early 2014, making it the 20th Gavi-eligible country and the 53rd country worldwide to include rotavirus vaccines into its immunization program. Our data highlight the speed with which this was done and the success of the outcome. By demonstrating that rotavirus vaccine coverage falls somewhat below target (84.3% at 9 months of age) at a time when changes in the supply chain are needed, and that coverage of measles and yellow fever vaccines remains unacceptably low at a time when active transmission is occurring,^29^ these surveys help to highlight priorities for continued strengthening of the Malian EPI.
